# Nocturnal sap flow as compensation for water deficits: an implicit water-saving strategy used by mangroves in stressful environments

**DOI:** 10.3389/fpls.2023.1118970

**Published:** 2023-05-08

**Authors:** Sipan Wu, Xiaoxuan Gu, Yanghang Zheng, Luzhen Chen

**Affiliations:** State Key Laboratory of Marine Environmental Science, Key Laboratory of the Ministry of Education for Coastal and Wetland Ecosystem, College of the Environment and Ecology, Xiamen University, Xiamen, Fujian, China

**Keywords:** mangrove, nocturnal sap flow, stem diameter change, stem water refilling, transpiration, vapor pressure deficit, water-saving mechanism, water-use strategy

## Abstract

As part of the plant water-use process, plant nocturnal sap flow (*Q*_n_) has been demonstrated to have important ecophysiological significance to compensate for water loss. The purpose of this study was to explore nocturnal water-use strategies to fill the knowledge gap in mangroves, by measuring three species co-occurring in a subtropical estuary. Sap flow was monitored over an entire year using thermal diffusive probes. Stem diameter and leaf-level gas exchange were measured in summer. The data were used to explore the different nocturnal water balance maintaining mechanisms among species. The *Q*_n_ existed persistently and contributed markedly over 5.5%~24.0% of the daily sap flow (*Q*) across species, which was associated with two processes, nocturnal transpiration (*E*_n_) and nocturnal stem water refilling (*R*_n_). We found that the stem recharge of the *Kandelia obovata* and *Aegiceras corniculatum* occurred mainly after sunset and that the high salinity environment drove higher *Q*_n_ while stem recharge of the *Avicennia marina* mainly occurred in the daytime and the high salinity environment inhibited the *Q*_n_. The diversity of stem recharge patterns and response to sap flow to high salinity conditions were the main reasons for the differences in *Q*_n_/*Q* among species. For *Kandelia obovata* and *Aegiceras corniculatum*, *R*_n_ was the main contributor to *Q*_n_, which was driven by the demands of stem water refilling after diurnal water depletion and high salt environment. Both of the species have a strict control over the stomata to reduce water loss at night. In contrast, *Avicennia marina* maintained a low *Q*_n_, driven by vapor pressure deficit, and the *Q*_n_ mainly used for *E*_n_, which adapts to high salinity conditions by limiting water dissipation at night. We conclude that the diverse ways *Q*_n_ properties act as water-compensating strategies among the co-occurring mangrove species might help the trees to overcoming water scarcity.

## Introduction

Daily water use strategy, reflected by both diurnal and nocturnal sap flow, is a critical component of plant water physiology (Steppe et al., 2006). Nocturnal sap flow is the night-time movement of fluid within the sapwood of root, stem or branch in trees (Forster, 2014). With the development of sap flow techniques, a substantial amount of evidence has shown that the nocturnal sap flow (*Q*_n_) occurs in a range of woody plants in different biomes and contributes significantly to daily sap flow (*Q*) (Dawson et al., 2007; Pfautsch et al., 2011; Forster, 2014). On a global scale, *Q*_n_ contributed over 12% of *Q* among various woody plants in different biotas and the *Q*_n_/*Q* can reach up to 69% in some species (Forster, 2014). The ubiquity of *Q*_n_ and its potentially high values have significant implications to model plant and ecosystem water and energy balances and need to be accurately quantified (Zeppel et al., 2010; Zeppel et al., 2014).

For terrestrial plants, *Q*_n_ consists of two significant ecophysiological and ecohydrological components: transpiration of water from the canopy and stem water refilling at night (Daley & Phillips, 2006; Caird et al., 2007; Forster, 2014). Both processes influence forest water budgets and plant responses to water stress (Zeppel et al., 2014; Chen et al., 2020). For example, continuous nocturnal transpiration helps transports oxygen and nutrients into the thin-walled cells of the plant xylem, which maintains the oxygen and nutrient requirements of the plant cells (Daley and Phillips, 2006). Nocturnal stem water refilling contributed to removing embolisms and repairing cavitation in eight plant species in mesic and semi-arid environments (Zeppel et al., 2019). Previous research has also shown that *Q*_n_ improved the pre-dawn disequilibrium relationship between soil and plant water potential in four temperate conifer species in northern Idaho (Kavanagh et al., 2007). The contribution of these two components to *Q*_n_ varies widely among species. Liu et al. (2021) found that *Q*_n_ in *Platycladus orientalis* and *Quercus variabilis* in a temperate forest consisted mainly of stem water refilling, which effectively maintained the stem water balance under drought stress. However, Zeppel et al. (2010) found that the nocturnal transpiration was the main component of *Q*_n_ in two co-occurring evergreen forest species, *Eucalyptus parramattensis* and *Angophora bakeri*. The ecophysiological controls over *Q*_n_ differed even under identical environmental conditions and different patterns of nocturnal water use also reflect habitat adaptation of species (Daley & Phillips, 2006). Therefore, quantifying the contributions of nocturnal transpiration and stem water refilling to *Q_n_*, has important implications in understanding nocturnal plant water-use strategies.

Growing evidence has suggested that in some plants the stomata remained open or partially open at night (Gindel, 1970; Caird et al., 2007; Fisher et al., 2007; Zhao et al., 2017), and that incomplete stomata closure provides the transpirational pull needed for nocturnal transpiration. Vapor pressure deficit (VPD) has been identified in some studies as the most crucial environmental driver of nocturnal transpiration (Dawson et al., 2007; Fisher et al., 2007; Zeppel et al., 2014; Wu et al., 2020). Other studies reported negative (Bucci et al., 2004) or negligible effects (Barbour et al., 2005) of VPD on nocturnal stomata. Wind speed affected the moisture movement around plant canopy (Fricke, 2019) and thus influenced on nocturnal water loss (Chen et al., 2020), which also could not be ignored. However, some studies discovered the opposite result that wind speed had little effects on the nocturnal water loss (Wang et al., 2018; Wu et al., 2020). Daytime water consumption during transpiration is also an important factor affecting *Q*_n_. Stem tissue water storage in is often depleted or partially depleted after intense transpiration during the daytime. This forces the roots to absorb water during the night to overcome the water deficit (Oren et al., 1999). At the end of the century, the predicted global average surface air temperature is projected to be 1.4 to 4.4°C higher than that in 1850–1900, with larger increases observed at night, especially in the tropics (IPCC, 2014; IPCC, 2021). Such a rise in temperature will result in an exponential climb in VPD, and more specifically in leaf-to-air VPD (Grossiord et al., 2020), which will affect plant transpiration during the day and enhance atmospheric evaporative demand at night (Zeppel et al., 2014). The processes of *E*_n_ and *R*_n_ will ultimately be affected. A better understanding of *Q*_n_ will help to provide accurate predictions of plant adaptation in the face of climate change.

Mangroves are salt-tolerant plants that grow in the intertidal zones of tropical and subtropical latitudes around the world (Tomlinson, 2016). The strong osmotic gradient between the plant and saline growing environment and the highly negative water potentials of soil pore water, making water acquisition more energetically unfavorable than in non-saline soils (Reef and Lovelock, 2014; Barraclough et al., 2020) and eventually lead to physiological water shortage of mangroves (Saenger, 2002; Lambs & Saenger, 2011). Furthermore, nocturnal stem water refilling would be less likely to occur under these conditions because it would take more transpiration pull to overcome this osmotic water potential gradient. Therefore, maintaining water balance is always a challenge (Barraclough et al., 2020). Water-use strategies are generally considered to be a key factor in the adaptation of mangroves to high saline intertidal conditions (Muller et al., 2009). Recent stable isotope studies have shown that mangroves utilize less saline water sources when freshwater is available (Wei et al., 2013). Scholander et al. (1962) found that mangroves overcome the high osmotic potential of saline environments through both osmotic and hydrostatic potential in their xylem sap. Previous studies demonstrated that mangroves adopt a conservative water-use strategy and use water efficiently during photosynthesis (Krauss et al., 2008; Krauss et al., 2015). Monitoring has shown that mangroves maintain a certain amount of sap flow at night (Leng & Cao, 2020). Waisel et al. (1986) showed that mangroves maintain low hydrostatic potential during nighttime, which would allow mangroves to overcome osmotic potential of the seawater in the root environment. For those mangrove species with conservative nocturnal water use, *Q*_n_ would be an adaptive strategy coping with temporary water deficit by effectively buffering stem water depletion after daytime transpiration. Mangroves grow in anoxic soil conditions (Lovelock et al., 2016), and *E*_n_ would also help maintaining cell oxygen. However, our knowledge of the nocturnal water use of mangroves is still limited. Understanding the physiological implications of *Q*_n_ in mangroves is very important for exploring the adaptability of mangroves to special intertidal habitats.

To better understand the water-use strategies of mangrove species during the night, we measured sap flow in three co-occurring mangrove species, *Kanderia obovata, Avicennia marina*, and *Aegiceras corniculatum*, over an entire year (2019). The mangroves were located in a subtropical estuary mangrove forest in the Fujian Province of China. Meteorological factors were measured synchronously. Diurnal variations in stomatal conductance and stem diameter of the three species were also measured on typical clear days in summer. The objectives of this study were to (1) quantify the contribution of *Q*_n_ to *Q* and its implication for water use, (2) explore the allocation and utilization patterns of *Q*_n_, and (3) identify the driving forces of *Q*_n_ in three co-occurring mangrove species. We expected to reveal a new mechanism for how mangroves maintain water balance during the night after experiencing high water consumption by transpiration during the daytime, and to better understand the nocturnal water-use strategies and the adaptability to their unique habitats of mangroves.

## Materials and methods

### Site description

Field measurements were conducted for one entire year (2019) in the Zhangjiang Estuary Mangrove National Nature Reserve (23.9240°N, 117.4147°E) in the Fujian Province of China. The annual weather conditions in the estuary followed the patterns typical of a subtropical monsoon climate. Daily mean relative humidity (RH) ranged from 24.2% to 100% with a mean of 83.2%. Annual temperature extremes ranged from 9.35°C to 37.9°C with an annual mean air temperature of 23.7°C. The annual precipitation was 1714 mm with the peak in spring and summer. The estuary experiences a regular semidiurnal tide with an annual mean tidal range of 2.32 m. The sediment surface in mangrove forests is normally flooded with a maximum inundation depth of around 1.0 m. Salinity fluctuates seasonally, with measured minimum values of approximately 13-18 practical salinity units (PSU) during November-December and maximum values of 3-13 PSU during June-September. The mangrove forest naturally consists of three co-occurring species, *Kandelia obovate* (dominant), *Avicennia marina*, and *Aegiceras corniculatum*. Four trees of each species in close proximity to one another were randomly selected for sap flow monitoring and related measurements. The morphological features of the trees were measured before sap flow monitoring (**Table 1**).

**Table 1:** Tree height, diameter at breast height (DBH), Sapwood depth, canopy projection area and sensor type of the trees randomly selected for sap flow monitoring (Mean ± s.e., n=4).

Species	n	Tree height (m)	DBH (cm)	Sapwood depth (cm)	Canopy projection area (m^2^)	Sensor type
*Kandelia*	4	5.3 ± 0.0	8.8 ± 0.7	4.1 ± 0.5	4.5 ± 0.6	TDP30
*Avicennia*	4	4.9 ± 0.1	7.4 ± 0.5	3.3 ± 0.2	3.3 ± 0.9	TDP30
*Aegiceras*	4	2.5 ± 0.2	3.4 ± 0.2	1.3 ± 0.3	1.0 ± 0.2	TDP10

The canopy projection area (m^2^) was calculated by an elliptic equation after measuring the canopy length (C*_l_*) and canopy width (C*_w_*) of each tree (Wang et al., 2018):


(Eqn. 1) $$Canopyprojection\;area=\frac{\pi \times C_{\text{l}}\times C_{\text{w}}}{4}$$

### Environmental factor monitoring

To investigate plant physiological responses to the microclimate, a weather station was installed in the study area. A sensor of HOBO MX2301 (Onset Computer Corp., Bourne, MA, USA) was installed on an Eddy tower above the canopy to monitor air temperature (T_a_, °C) and relative humidity (RH, %). Vapor pressure deficit (VPD, kPa) was calculated from T_a_ and RH. Photosynthetically active radiation (PAR) was measured from the data collected on an Eddy tower above the canopy using a PQS1 PAR Quantum sensor (Kipp & Zonen). Wind speed were measured using a 010C Wind speed Sensor (Met One Instruments, Inc) made above the mangrove canopy. Meteorological data were recorded every 30 s and stored at 10 min average in a data logger (CR1000 datalogger, Campbell Scientific, Inc., Logan, UT, USA). Tidal surface water salinity was also continuously measured and estimated from conductivity and temperature measurements using HOBO U24-002-C Conductivity Logger (Onset) deployed just above sediment surface. Raw 10-minute tidal data were consistently converted to half-hourly time series for data analysis.

### Sap flow measurements

**Figure 1:**
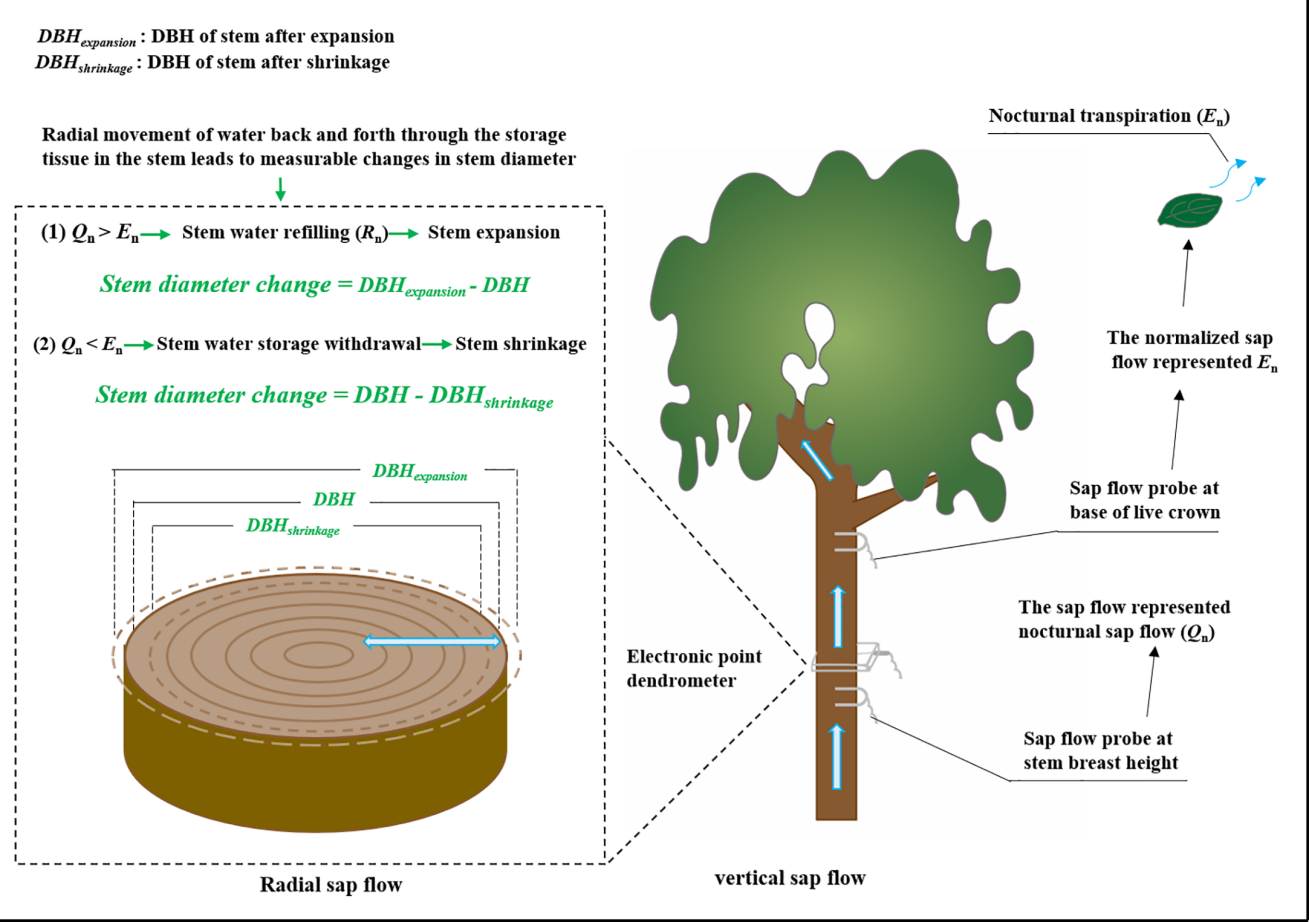
Schematic diagram of monitoring of sap flow and stem diameter change.


Sap flow was measured using Granier-type thermal dissipation probes as per Zhao et al. (2018) (**Figure 1**). In order not to damage the sample trees, we selected 4 trees of different diameter classes in each of the three species to establish the relationship between diameter at breast height and sapwood depth, and the operation method referred to the sapwood measurement method mentioned by Leng & Cao, 2020. Two different size probes (TDP10 with a probe length of 10 mm, and TDP30 with a length of 30 mm) were used to fit different sapwood widths. Each probe was installed at 1.3 m above the sediment surface and on the north-facing side of the stem to avoid the effects of sunlight. The probes were covered with reflective aluminum sheets and wrapped with a plastic tarp to protect them from solar irradiation and rain damage. Each probe consisted of two needles with thermocouples and a heater. The upper needle was continuously heated with a constant power of 0.2 W, while the lower needle stayed at the temperature of the unheated sapwood. The temperature difference between the two probes was measured at 30 s intervals, and the averaged values for each 10 min were recorded with a data logger (CR1000, Campbell Scientific Inc., Logan, UT, USA). The monitoring period was from January 1 to December 31, 2019. The sap flux density (SFD, g cm^−2^ s^−1^) was calculated based on the differential temperature (Δ*T*, °C) between the heated and reference probes following an empirical equation (Granier, 1987):

(Eqn. 2) $$SFD=\;119\;\times {\left(\frac{\Delta T_{\text{m}}-\Delta T}{\Delta T}\right)}^{1.231}$$

where Δ*T* is the measured differential temperature, and Δ*T*_m_ represents the Δ*T* when Sap flow density is zero.

The xylem wood of the three species in this study is diffuse porous (Lin et al., 1998). According to previous studies, the radial sap flow density is not significantly different in diffuse-porous wood (Phillips et al., 1996; Clearwater et al., 1999). Therefore, the daily sap flow (*Q*, kg d^−1^) for each sampled tree was calculated as follows:


(Eqn. 3) $$Q=\sum \left(SFD\times A_{s}\times t\right)$$

where *A_s_* is the cross-sectional sapwood area of an individual tree, and *t* is 10 min because the data are being stored as 10 min averages.

### Differentiation of *E*_n_ and *R*_n_

To quantify the contribution of the two components (*E*_n_ and *R*_n_) to the *Q*_n_, the sap flow of three sample trees of each species was monitored at breast height and the base of the live crown (**Figure 1**) (Daley & Phillips, 2006). In this analysis, the 24-h sum of sap flow at the base of the live crown was assumed to be equal to the 24-h sum of sap flow at breast height (Goldstein et al., 1998; Daley & Phillips, 2006). We normalized the 24-h sum of sap flow at the base of the live crown in each sample tree so that it equaled the 24-h sum of the sap flow at breast height. Specifically, 10-min sap flow at the base of the live crown multiply the ratio of the 24-h sum of sap flow at breast height to 24-h sum of sap flow at the base of the live crown. This is similar to the approach described by Goldstein et al. (1998). Previous studies showed lower water storage in the crown than in the stem (Waring et al., 1979), so we assumed the normalized sap flow at the base of the live crown could represent the total transpiration (Daley & Phillips, 2006). We calculated the difference between the sap flow rates at the stem breast height and mean transpiration at 10-min intervals. The positive values represented stem water refilling, whereas the negative values represented stem water storage was used by transpiration (Goldstein et al., 1998; Daley & Phillips, 2006; Zeppel et al., 2010).

### Stem diameter variations measurements

Since their first appearance in 1970, point or ring dendrometers have been increasingly used for the measurement of stem diameter variation. These first observations suggest a close link between diurnal variation in plant water status and diurnal variation in stem thickness. Radial movement of water back and forth through the storage tissue in the stem leads to measurable changes in stem diameter (Barraclough et al., 2020). The stem diameter variations were measured with precision dendrometers (**Figure 1**). To explore the radial movement of water in the stem, electronic point dendrometers (RRDIM10, Rainroot, China) were installed at the breast height of the main stem on two of the four sampled trees of each species. The resolution of the electronic point dendrometers was 10 μm. Data were obtained every 15 s and instruments were logged at 5 min intervals with a data logger (RR-1004, Rainroot, China). To get the largest diurnal variations of stem diameter during the same measuring period, stem diameter was measured during seven typical clear days in summer (from August 22 to August 28, 2019). We took the stem diameter value at 0:00 on a day after the equipment was stabilized as the stable point of reference.

### Leaf gas exchange measurements

Leaf transpiration (*T_r_*) and stomatal conductance (*g_s_*) from three trees of each species were measured on a typical sunny day in summer (August 26, 2019). Three to five mature, healthy, and fully expanded leaves on a south-facing branch were randomly selected from lower and upper crown locations for leaf gas exchange measurement using a portable photosynthesis system (Li-6400xt, Li-cor, USA) (**Figure 1**). Measurements were taken at 2-hr intervals for 24 hours from 10:00 on the first day to 10:00 on the following morning. During the measurements, PAR, T_a_, RH, and CO_2_ concentrations in the leaf chamber were set to match ambient environmental conditions. The measurement chamber was not illuminated at night.

### Data analysis and statistics

Night was defined as the period from 18:00 to 6:00 on the following day with PAR lower than 20 μmol m^−2^ s^−1^ (Daley & Phillips, 2006; Wang et al., 2011; Siddiq & Cao, 2018). We subdivided night into a first part (18:00 to 24:00) and second part (0:00 to 6:00). To calculate the amount of stem expansion and shrinkage during the night, we averaged stem diameter data every 10 minutes (*D_i_*, where 1 ≤ *i* ≤ 72). When *D_i+1_ - D_i_* > 0, the stem was expanding and when *D_i+1_ - D_i_*_<_ 0, the stem was shrinking. Thus, the amount of stem expansion and shrinkage during the nighttime was calculated as follows:


(Eqn. 4) $$D_{i+1}-D_{i}>0:D_{e}=\sum^{\;}(D_{i+1}-D_{i})$$

(Eqn. 5) $$D_{i+1}-D_{i}<0:D_{s}=\sum \left(\left|\left(D_{i+1}-D_{i}\right)\right|\right)$$

where *D_i_* is the mean stem diameter at the i^th^ 10 minutes, and *D_i+1_* is the mean stem diameter value at the (i+1)^th^ 10 minutes. *D*_e_ is the cumulative amount of stem expansion and *D*_s_ is the cumulative amount of stem shrinkage.

The vapor pressure deficit (VPD, kPa) was determined using measured atmospheric temperature (T_a_) and relative humidity (RH), and the calculation formula is as follows (Buck, 1981):


(Eqn. 6) $$VPD=0.611\times \text{Exp}\left(\frac{17.27\times {\text{T}}_{\text{a}}}{T_{a}+237.3}\right)\times \left(1-RH/100\right)$$

Since the sap flow data for the entire year did not have a normal distribution, we used the nonparametric Kruskal-Wallis test to analyze the difference between *Q*, *Q*_n_, and the contribution of *Q*_n_ to *Q* (*Q*_n_/*Q)* among the three mangrove species. An independent sample *t*-test was conducted to test whether there were significant differences between the mean cumulative amount of stem expansion and the mean cumulative amount of stem shrinkage for each species. The general linear regression and curvilinear model regression were fitted to identify the relationships between (1) salinity and sap flow (daily sap flow and nocturnal sap flow), (2) sap flow density (SFD) (daytime SFD, nocturnal SFD, SFD in the first part of the night and SFD in the second part of the night) and VPD, (3) wind speed sap flow (daily sap flow and nocturnal sap flow), (4) *Q*_n_ and *Q*. At the same time, the analysis of Covariance (ANCOVA) was used to test whether there were significant differences in the slopes of fitted linear relationships for different species within the same salinity environment. The Gompertz models (three parameters) was used to test the relationship between the *Q*_n_/*Q* of different tree species and latitude. The *Q*_n_/*Q* data (n=56) for different tree species were based on data provided by Forster (2014). The significance level was set at *P<* 0.05. All data were analyzed using SPSS 25.0 (SPSS, Chicago, Illinois, USA).

## Results

### Magnitude of nocturnal sap flow in mangrove forests

**Figure 2:**
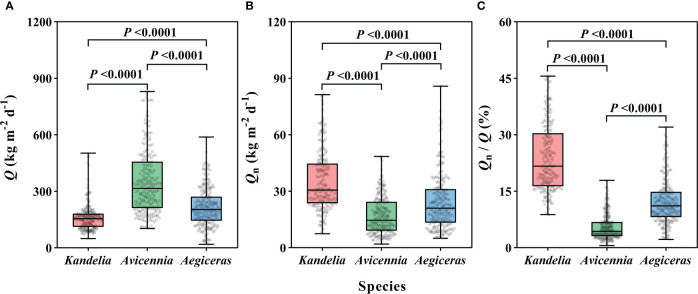
The daily sap flow (*Q*) in per sapwood area **(A)**, the nocturnal sap flow (*Q*_n_) **(B)**, and the proportion of *Q*_n_/*Q* **(C)** of each species from January to December, 2019. Data of the three species were retrieved from the same five consecutive typical sunny days per month. Statistical results from Kruskal-Wallis test in nonparametric tests among the three species were shown. For each species, the values reported were from four replicated trees. Significance level was set at *P<* 0.05.

For the species *Kandelia obovata*, *Avicennia marina*, and *Aegiceras corniculatum*, the annual mean *Q* per sapwood area ranged from 162.0 Kg m^−2^ d^−1^ to 348.1 Kg m^−2^ d^−1^ (**Figure 2A**). The magnitude of the annual mean *Q*_n_ per sapwood area varied from 16.9 ± 0.7 Kg m^−2^ d^−1^ to 35.2 ± 1.1 Kg m^−2^ d^−1^ (**Figure 2B**). The annual mean *Q*_n_/*Q* was 24.0 ± 0.6%, 5.5 ± 0.2%, and 12.1 ± 0.4% for *Kandelia obovata*, *Avicennia marina*, and *Aegiceras corniculatum*, respectively. These values were up to 45.6%, 17.9% and 32.0%, respectively, higher on some days (**Figure 2C**). For *Q*, *Q*_n_, and *Q*_n_/*Q*, significant differences were observed among the three species. The *Q* of *Avicennia marina* was significantly higher than the *Q* of the other two species, while the *Q*_n_ of *Avicennia marina* was significant lower than the *Q*_n_ of the other two species (**Figures 2A–C**).

### Nocturnal leaf stomatal conductance and transpiration rate

**Figure 3:**
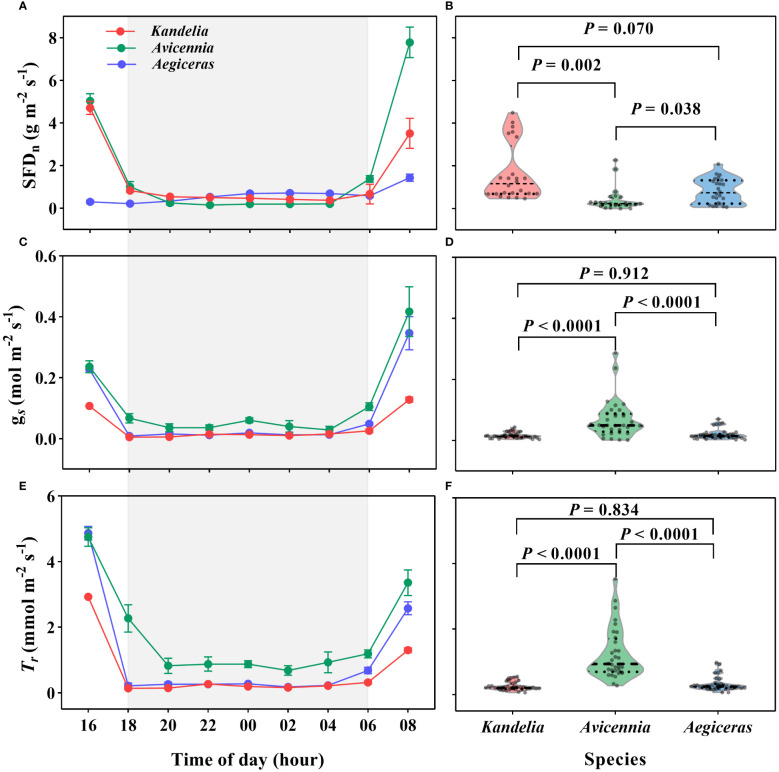
The variation patterns of the nocturnal sap flow density (SFD_n_) at breast height **(A, B)**, leaf-level stomatal conductance [*g_s_*, **(C, D)**] and leaf-level transpiration rate (*T_r_*, E and R) of the three species during the nighttime on a typical sunny day (26 August 2019). Statistical results from One-Way ANOVA among the three mangrove species were shown. Each SFD values for each species were the mean of four replicated trees. Significance level was set at *P*< 0.05. The shaded area in **(A, C, E)** represented the nighttime.

A substantial amount of SFD and leaf *T_r_* was detected during the night. For all three species, SFD, leaf *T_r_* and *g_s_* decreased sharply after 16:00 and then remained steady until sunrise on the following day (**Figures 3A, C, E**). During the night, *Kandelia obovata* and *Aegiceras corniculatum* maintained low *g_s_*, less than 0.02 mol H_2_O m^−2^ s^−1^. These values decreased to about 9% and 6%, respectively, of day values. *T_r_* was also low, below 0.30 mmol H_2_O m^−2^ s^−1^ (**Figures 3C, E**). There was no significant difference in *g_s_* and *T_r_* between *Kandelia obovata* and *Aegiceras corniculatum* (**Figures 3D, F**). Although the nocturnal SFD of the *Avicennia marina* was significantly lower than that of the other two species, the *g_s_* and *T_r_* were significantly higher (**Figures 3B, D, F**). The mean nocturnal *g_s_* of *Avicennia marina* was 0.05 mol H_2_O m^−2^ s^−1^, and the value decreased to 16% of day value. The mean nocturnal *T_r_* of *Avicennia marina* was 1.09 mmol H_2_O m^−2^ s^−1^ (**Figures 3C, E**).

### Nocturnal transpiration and stem water refilling

**Figure 4:**
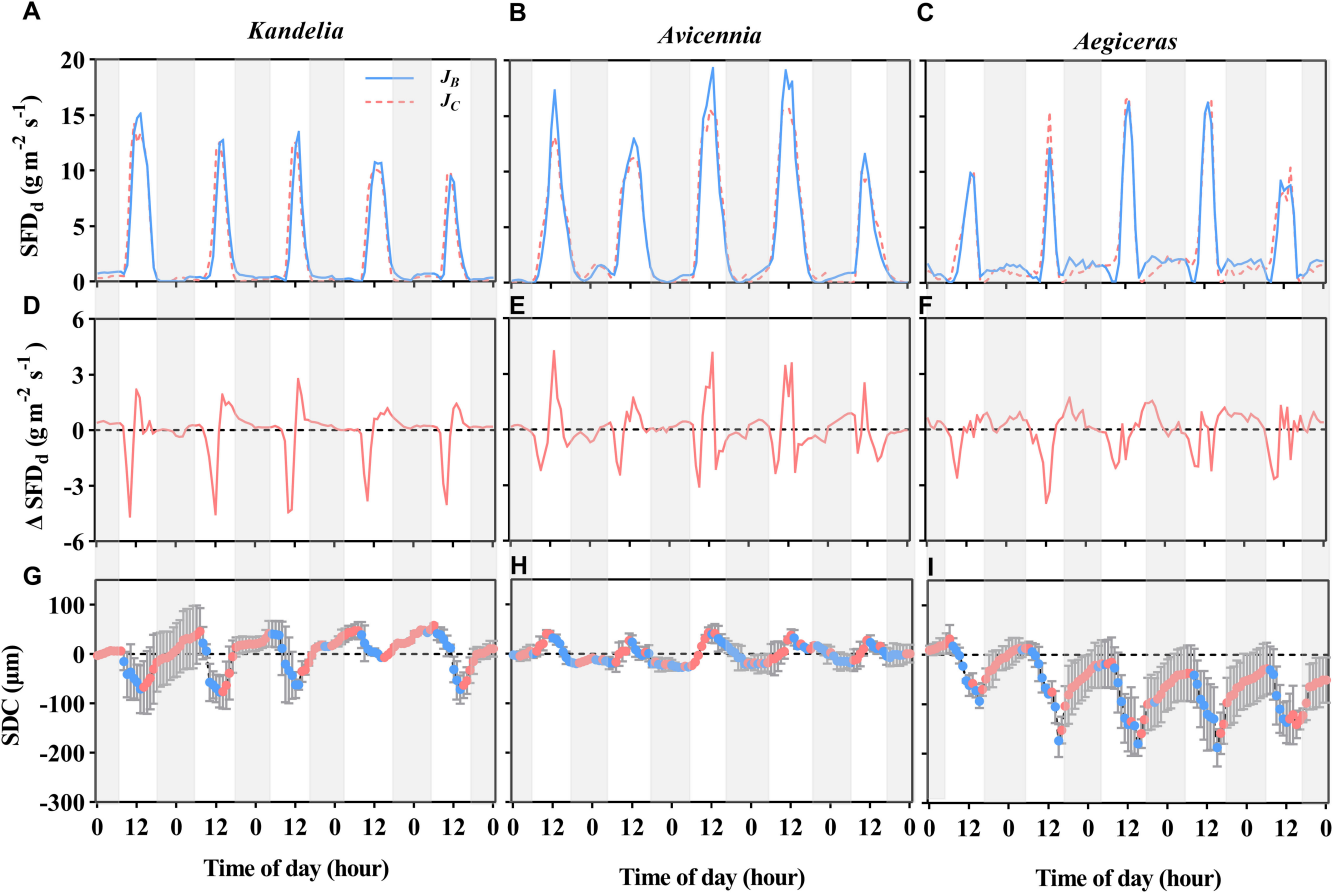
The variation patterns of the diurnal sap flow density (SFD_d_) of three mangrove species **(A–C)** within the same five consecutive typical sunny days (from Aug. 24 to Aug. 28 in 2019), and the difference between breast height sap flow density (*J_B_*) and the normalized sap flow density at the base of the live crown (*J_C_*) [Δ SFD_d_, **(D–F)**]. Positive value in the **(D–F)** indicate time periods when stem water recharge, and negative values indicate time periods when canopy transpiration was preferentially stem water storage. The diurnal patterns of stem diameter change (SDC) for each species [**(G–I)**, the red circles represent stem expansion and the blue circles represent stem shrinkage]. The shaded area represents nighttime.

Early in the morning (about 6:00-10:00), sap flow at breast height was lower than sap flow at the base of the live crown, indicating that the three mangrove species utilized water stored in the stem for transpiration at sunrise (**Figures 4A–C**). Stem water refilling and stem water storage withdrawal during the night occurred in all three species, but stem water refilling occurred most of the night for *Kandelia obovata* and *Aegiceras corniculatum*, while *Avicennia marina* experienced mainly stem water storage withdrawal (**Figures 4D–F**). The contribution of *E*_n_ and *R*_n_ to *Q*_n_ in *Kandelia obovata* was 43.7% and 56.3%, respectively. Similar to *Kandelia obovata*, the contribution of *E*_n_ and *R*_n_ to *Q*_n_ in *Aegiceras corniculatum* was 32.7% and 67.3%, respectively. In contrast, the *Q*_n_ of *Avicennia marina* consisted mainly of *E*_n_ and the *E*_n_ exceeded the *Q*_n_ (*E*_n_ accounted for 103% of *Q*_n_). In *Avicennia marina*, stem water refilling occurred during the day and stem water storage was partially depleted at night during the nocturnal transpiration process (**Figure 4E**).

### Stem diameter change patterns

**Figure 5:**
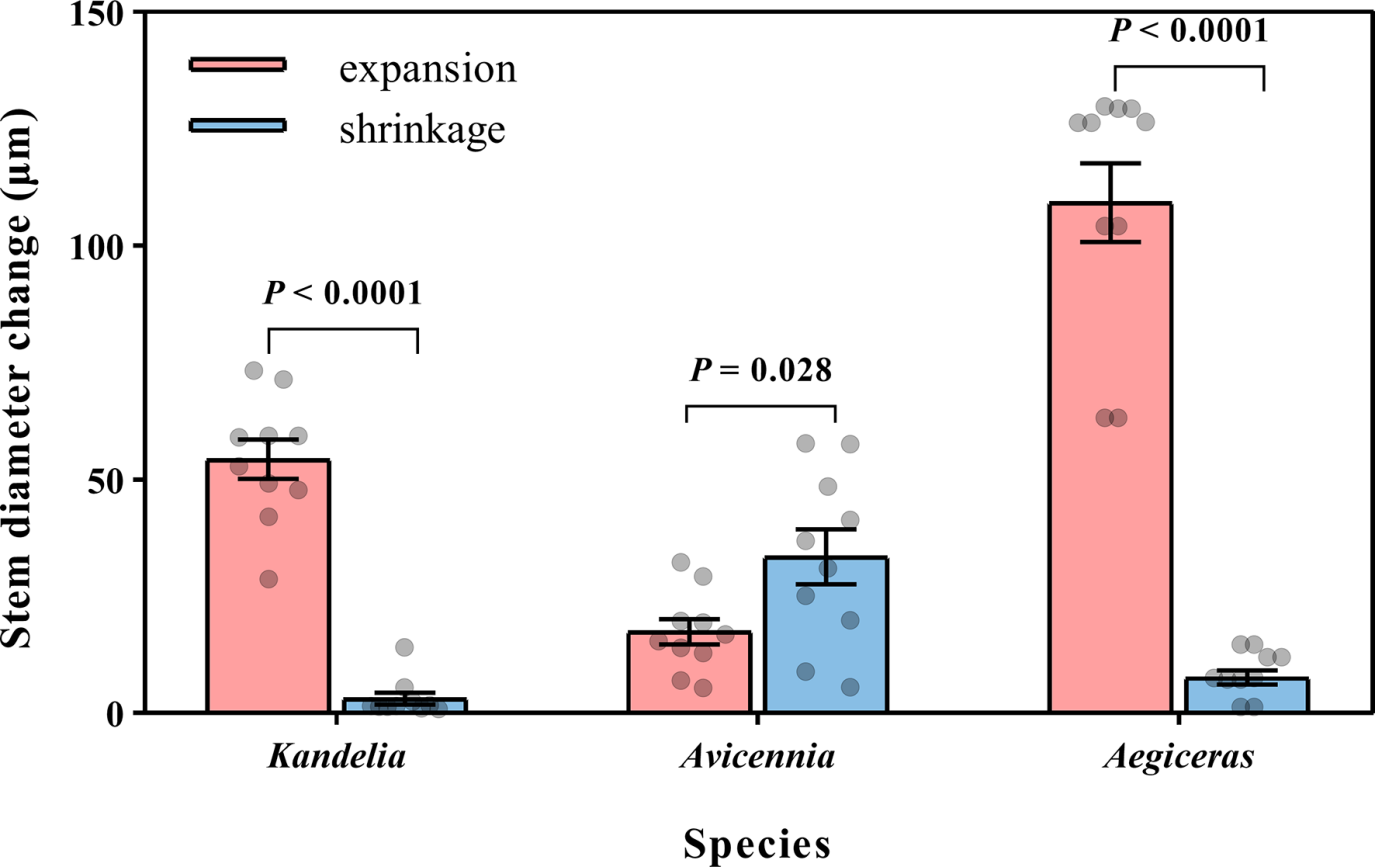
Mean night-time cumulative amount of stem expansion and shrinkage of three mangrove species within the same five consecutive typical sunny days (from Aug. 24 to Aug. 28 in 2019). Significant differences between expansion and shrinkage was analyzed by independent sample t-test. For each species, the values reported were from two replicated trees. Significance level was set at *P<* 0.05.

For *Kandelia obovata* and *Aegiceras corniculatum*, stem diameter decreased in the morning and started to increase again in the afternoon. Although the increasing trend of stem diameter in *Kandelia obovata* and *Aegiceras corniculatum* at night was weaker than the increasing trend before the sunset, the stem diameter of these two species remained in an expanded state at night (**Figures 4G, I**). However, the stem diameter changes of *Avicennia marina* showed different diurnal patterns from the former two species, with an increase during the morning and a decrease in the afternoon, which follow the variation of the SFD (**Figure 4H**). Unlike *Kandelia obovata* and *Aegiceras corniculatum*, little the cumulative amount of stem expansion can be detected during certain nighttime periods in *Avicennia marina*. By the independent sample *t*-test analysis, the cumulative amount of stem expansion was significantly higher than the cumulative amount of stem shrinkage in *Kandelia obovata* and *Aegiceras corniculatum.* However, the cumulative amount of stem expansion was significantly lower than the cumulative amount of stem shrinkage in *Avicennia marina* (**Figure 5**).

### Relationships between salinity and sap flow

**Figure 6:**
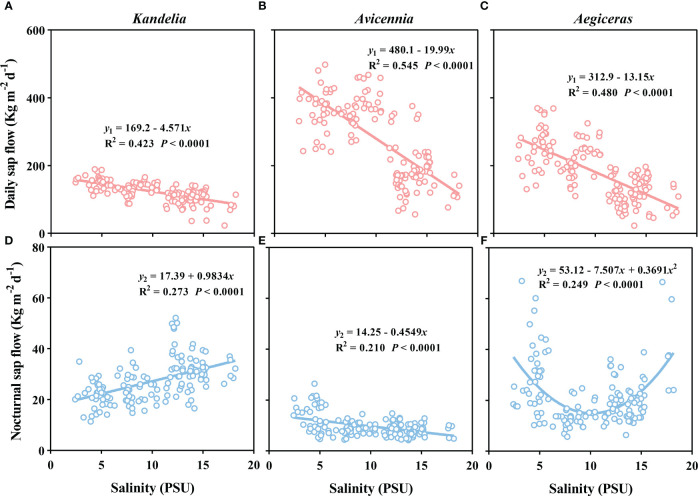
The relationship between salinity and daily sap flow **(A–C)**, and the relationship between salinity and nocturnal sap flow **(D–F)** in per sapwood area for *Kandelia*, *Avicennia* and *Aegiceras* from August to December.

There was a significant negative linear relationship between the salinity and the daily sap flow of the three species (**Figure 6**). The slopes of fitted linear relationships between salinity and daily sap flow of *Kandelia obovata* was significantly lower than that of *Aegiceras corniculatum* and *Avicennia marina*. When the ambient salinity increased from 5 PSU to 15 PSU, the daily sap flow of *Kandelia obovata*, *Avicennia marina* and *Aegiceras corniculatum* decreased to 26.3%, 48.3% and 50.6%, respectively. During the night, different species showed different relationship between salinity and sap flow. There was a positive linear relationship between salinity and sap flow of *Kandelia obovata* while a negative linear relationship between salinity and sap flow of *Avicennia marina* (**Figures 6A, B**). For *Aegiceras corniculatum*, there was a non-linear relationship between salinity and sap flow (**Figure 6C**).

### Relationships between wind speed, VPD, and sap flow

**Figure 7:**
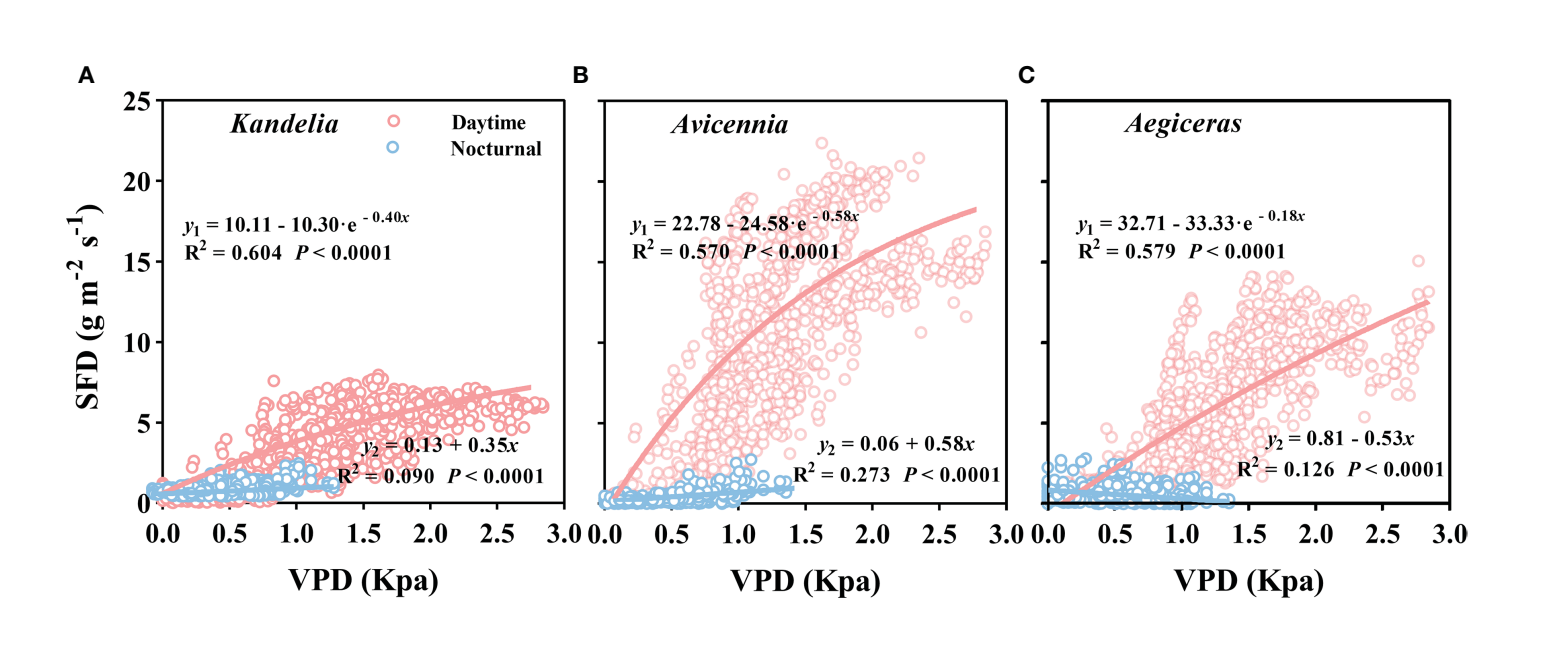
The relationship between vapor pressure deficit (VPD) and mean hourly sap flow density (SFD) for *Kandelia* **(A)**, *Avicennia* **(B)** and *Aegiceras* **(C)** from January to December. y1 and y2 represented daytime SFD and nocturnal SFD respectively. Data of each species were retrieved from the typical sunny days per month. The best fit exponential relationship (y = i + a(1-e-bVPD)) is shown for daytime (red line) and linear relationship (y = i + a·VPD) was shown for nocturnal (blue line).

As can be seen from **Figure S1**, wind speed had no significant effect on the daily sap flow and nocturnal sap flow. The relationships between SFD and VPD differed between day and night. During the day, there was an exponential relationship between SFD and VPD (SFD = *i* + a · (1 - e^-bVPD^)), and the VPD explained 57.0%–60.4% SFD variations in three mangrove species. During the night, a weak generally linear relationship (SFD = *i* + a · VPD) existed (**Figure 7**). Furthermore, the correlation coefficient (R^2^) differed among the three mangrove species (**Figure 7**). For *Avicennia marina*, the VPD explained 27.3% of the nocturnal SFD variation, whereas for *Kandelia obovata* and *Aegiceras corniculatum*, it explained only 9.0% and 12.6%, respectively (**Figures 7A–C**). SFD presented a sharp decreasing trend after sunset in *Kandelia obovata* and *Avicennia marina*, which was similar to the change of VPD. The SFD of *Kandelia obovata* showed a slow declining trend after 20:00 and maintained a high SFD until sunrise of the next day. After a sharp decline, the SFD of *Avicennia marina* stabilized and remained low in the second part of the night (*N*_s_). In contrast, the SFD of *Aegiceras corniculatum* showed an increasing trend after a brief decline at night, and then maintained a steady-state with high SFD in *N*_s_. The variation in nocturnal SFD of *Aegiceras corniculatum* was inconsistent with the variation of VPD (**Figure S2**). For *Kandelia obovata* and *Avicennia marina*, the responses of the nocturnal SFD to VPD occurred in the first part of the night (*N*_f_). VPD explained 21.1% and 50.3% of the nocturnal SFD variation, respectively. Interestingly, there was a significant negative correlation between the nocturnal SFD and VPD of *Aegiceras corniculatum* (**Table 2**).

**Table 2:** Liner regressions of nocturnal sap flow density (SFD_n_, g m^-2^ s^-1^) at breast height and vapor pressure deficit (VPD, Kpa) during the first part of the nighttime (*N*_f_, 18:00 - 24:00) and second part of the nighttime (*N*_s,_ 0:00 - 6:00), respectively.

Mode	Linear equation	*P* value	*R^2^*
*Kandelia - N*_f_	SFD_n_ = 0.311 + 0.502**×**VPD	**< 0.0001**	0.211
*Kandelia - N*_s_	SFD_n_ = 0.372 + 0.021**×**VPD	0.758	0.001
*Avicennia - N*_f_	SFD_n_ = -0.156 + 1.276**×**VPD	**< 0.0001**	0.503
*Avicennia - N*_s_	SFD_n_ = 0.162 + 0.046**×**VPD	0.143	0.027
*Aegiceras - N*_f_	SFD_n_ = 0.524 - 0.232**×**VPD	**0.004**	0.076
*Aegiceras - N*_s_	SFD_n_ = 1.047 - 0.797**×**VPD	**< 0.0001**	0.368

partial regression P value in bold are significant (P< 0.05).

### Relationships between *Q*_n_ and *Q*

**Figure 8:**
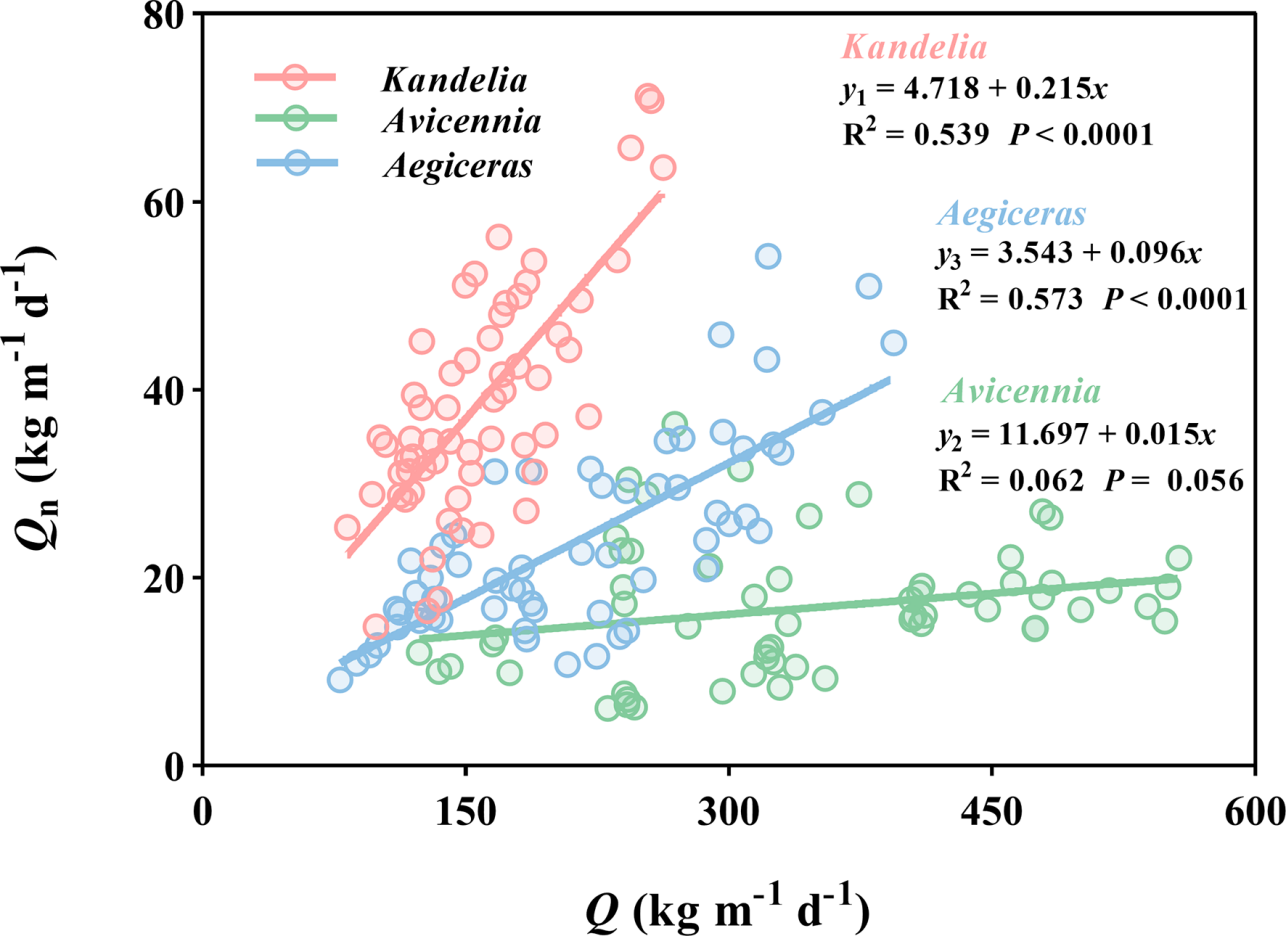
Linear relationships of daily sap flow (*Q*) and nocturnal sap flow (*Q*_n_) of the three mangrove species of the whole year. Data of each species were retrieved from the same five consecutive typical sunny days per month. For each species, the sap flow values reported were the mean of four replicated trees. *y*_1,_ *y*_2_ and *y*_3_ represented the *Q*_n_ of *Kandelia*, *Avicennia* and *Aegiceras* respectively.

The general linear regression analysis showed a significant and positive relationship between *Q*_n_ and *Q* in *Kandelia obovata* (*y =* 4.718 + 0.215*x*, R^2 = ^0.539, *P<* 0.0001) and *Aegiceras corniculatum* (*y =* 3.543 + 0.096*x*, R^2 = ^0.573, *P<* 0.0001). These results indicate that the *Q*_n_ increased with the increasing water lost from transpiration during the day. However, this linear relationship was not found between *Q*_n_ and *Q* of *Avicennia marina* (R^2 = ^0.062 P = 0.056) (**Figure 8**), and the *Q*_n_ of *Avicennia marina* was almost not affected by the water lost from transpiration during the day.

### Relationships between *Q*_n_/*Q* and latitude

**Figure 9:**
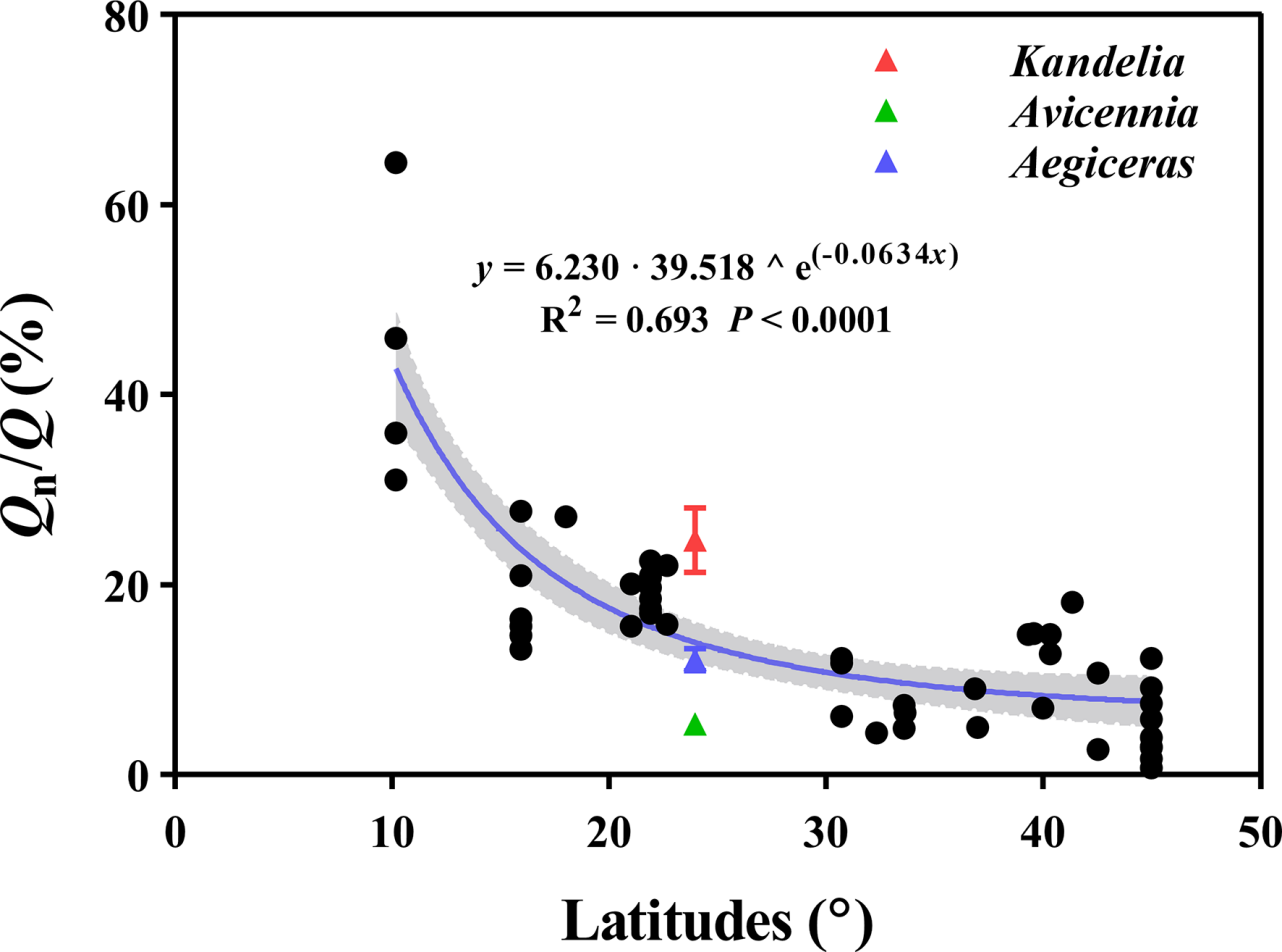
The relation between the *Q*_n_/*Q* of different tree species and latitudes. The data (n=56) of *Q*_n_/*Q* are mainly based on the data provided by Forster (2014). The red, green, and blue triangles represent *Kandelia*, *Avicennia* and *Aegiceras* in this study respectively. The shaded areas are represented by 95% confidence interval.

The curvilinear model regression analysis showed a significant and positive relationship between the *Q*_n_/*Q* of different tree species and latitudes (*y* = 6.203 · 39.518 ^ e^(-0.0634^ *^x^* ^)^, R^2 = ^0.693, *P<* 0.0001) (**Figure 9**).

## Discussion

### *Q*_n_/*Q* for mangrove species

Previous studies confirmed that *Q*_n_ occurs in species across all vegetation types and biomes, making a significant contribution to *Q* (Daley & Phillips, 2006; Zeppel et al., 2010; Forster, 2014). By analyzing 98 tree species from different biomes, Forster (2014) reported that *Q*_n_/*Q* was 7.6%, 16.5%, and 29.1% for forests in warm temperate, tropical, and equatorial, respectively. Our results demonstrated that *Q*_n_ occurred in each of the three mangrove species during most of the night. *Q*_n_/*Q* ranged from 5.5% to 24.0% of these subtropical mangrove species (**Figure 2C**). In the dataset of Forster (2014), a significant negative relationship (y = 6.230 · 39.518 ^ e^(−0.0634^ *^x^* ^)^) was observed between *Q*_n_/*Q* and latitude (**Figure 9**). The highest *Q*_n_/*Q* occurred in equatorial and tropical biomes, under conditions of high atmospheric evaporative demand, suggesting that tree species growing at lower latitudes would have higher *Q*_n_/*Q* (**Figure 9**). We predict that mangrove species growing in equatorial and tropical regions have even greater *Q*_n_, which has significant implications for the water balance between soil and plant and hydrology.

This study is one of the few to quantify inter-species differences of *Q*_n_ among the co-occurring species. Previous studies showed that *Q*_n_ rates varied among plant species because of different adaptive strategies (Phillips et al., 2010; Zeppel et al., 2010). In this study, significant differences were observed in the *Q*_n_/*Q* between species (**Figure 2C**), and this result supports the conclusion that different adaptive strategies lead to *Q*_n_/*Q* diversity. The relatively high average *Q*_n_/*Q* of *Kandelia obovata* and *Aegiceras corniculatum* in this study, indicating that the two species were highly dependent on *Q*_n_ and *Q*_n_ was important for maintaining water balance in these species. In contrast, *Avicennia marina* had little dependence on *Q*_n_. The reason for the difference in *Q*_n_/*Q* between species may be that stem water refilling of *Kandelia obovata* and *Aegiceras corniculatum* occurred mainly after sunset while the stem water refilling of *Avicennia marina* occurred during the daytime (**Figures 4D, E, F**). Due to the special habitat of mangrove species, we had to consider the effect of salinity changes on sap flow. For the *Q* of the three mangrove species, high salt conditions contribute to more conservative water use in mangroves (**Figure 6**). During the nighttime, high salt conditions drove high *Q*_n_ of *Kandelia obovata* and *Aegiceras corniculatum* while the high salt conditions inhibited the *Q*_n_ of *Avicennia marina*. The high salt conditions drove high *Q*_n_ may be related to mangrove species overcoming the high osmotic potential of saline environments through enhance both osmotic and hydrostatic potential in their xylem sap during the nighttime. The various responses of different species to salinity at night were also a reason for their different *Q*_n_/*Q*.

### Allocation and utilization patterns of *Q*_n_ for mangrove species

*Q*_n_ was associated with two physiological processes, *E*_n_ and *R*_n_. Direct evidence for *E*_n_ comes from leaf gas exchange measurements (Daley & Phillips, 2006). A previous study showed that weak stomatal conductance at night might result from slight *E*_n_ (Rosado et al., 2012). However, some reported not low levels of *Q*_n_ with incomplete stomatal closure during the night in many plant species (Snyder et al., 2003; Dawson et al., 2007). For *Kandelia obovata* and *Aegiceras corniculatum*, the SFD at night remained at a relatively high level with a low *g_s_* and *T_r_*, indicating that both species occurred weak *E*_n_ and had strict control over their stomata to reduce the water loss at night. In contrast, the *g_s_* and *T_r_* of *Avicennia marina* at night were significantly higher with lower SFD than the other two species (**Figures 3B, D, F**), indicating that a large part of the *Q*_n_ of *Avicennia marina* was dissipated into the atmosphere through the stomata. These different controls of leaf stoma in co-occurring species of the same environment were also reported in a mixed deciduous forest (Daley & Phillips, 2006).

The water-use pattern in which trees use stem stored water during the day to buffer water deficits and refill the stem water during the night time when evaporative demand is low, has been observed in different tree species (Goldstein et al., 1998; Phillips et al., 2003). Here, we found that in *Kandelia obovata* and *Aegiceras corniculatum*, *R*_n_ was a major component (over 60%) of *Q*_n_. The high ratio of *R*_n_ to *Q*_n_ indicates a strong reliance of these plants on stem storage water, which helps them to maintain a balance between water supply and water demand during periods with high transpiration demands (Scholz et al., 2011; McCulloh et al., 2014). High salinity conditions promote more conserved daytime water use in *Kandelia obovata* and *Aegiceras corniculatum* and drive higher levels of *Q*_n_, which maintains water balance in mangroves in highly negative water potential environments in the form of *R*_n_. Therefore, we deemed *Q*_n_ to be an effective water-use strategy to avoid physiological dehydration and adapt to high salt environments in some mangrove species. Unlike *Kandelia obovata* and *Aegiceras corniculatum*, the *Q*_n_ of *Avicennia marina* was mainly used for *E*_n_, and the stored water was released from the stem to supply the water needed for *E*_n_, indicating that the water consumption during transpiration at night was higher than the water absorbed by the roots from the soil. This leads to the question of how *Avicennia marina* maintained water balance at night after experiencing high water consumption by transpiration during the day. Different from *Kandelia obovata* and *Aegiceras corniculatum*, *Avicennia marina* is able to maintain water refilling while transpiring and uptake water from the saline soil more efficiently during daytime (**Figure 4E**). This process enables the water storage tissue to retain sufficient water after the water loss during the day and beneficial for maintaining stem water balance at night. Furthermore, previous studies showed that *Avicennia marina* had unique wood anatomy in which successive or multiple cambia resulted in consecutive bands of xylem interspersed with internal secondary phloem strands connected by parenchyma tissue (Schmitz et al., 2008; Robert et al., 2011). This anatomy is conducive to internal water storage and security and is deemed to be an adaptive trait to deal with physiological drought (Robert et al., 2011). At the same time, our results showed that *Avicennia marina* would limit *Q*_n_ under high salt conditions to reduce water loss at night, which was also a strategy to overcome physiological dehydration. In addition, Coopman et al. (2021) found that *Avicennia marina* can absorb atmospheric moisture through deliquescence of salt on leaf surfaces, driving top-down sap flow and replenishes the water needs of the stem. This may be one of the mechanisms for maintaining the water balance of *Avicennia marina* at night. Due to technical limitations, however, the thermal dissipation probe could not detect this part of the reverse flow. The neglect of reverse flow leads to an underestimation of its nocturnal sap flow and this will be a limitation of the experiment.

The changing patterns of stem diameter indirectly support our findings. From hourly to daily scale, the fluctuations in stem diameter reflected changes in the elastic tissue water content (Zweifel et al., 2001; Pfautsch et al., 2015). The cumulative amount of stem expansion was significantly higher than the cumulative amount of stem shrinkage in *Kandelia obovata* and *Aegiceras corniculatum* (**Figure 5**), which indicated that *R*_n_ occurred more frequently than stem water storage withdrawal at night. In contrast, the cumulative amount of stem expansion was significantly lower than the cumulative amount of stem shrinkage in *Avicennia marina* (**Figure 5**), indicating that relatively large of the stored water in the stems was released into the xylem flow to participate in the *E*_n_. The unusual stem variation pattern of *Avicennia marina* has also been reported by Barraclough et al. (2020).

The partitioning method used in this study had its limitations because the sap flow in terminal branches could not be measured, the same as the previous study (Daley & Phillips, 2006). For *Kandelia obovata* and *Aegiceras corniculatum*, the nocturnal *g_s_* and *T_r_* were low (**Figures 3D, F**), and SFD was low sensitive to VPD at night (**Figure 7**, **Table 2**), leading to low *E*_n_. Therefore, the contribution of *E*_n_ to *Q*_n_ may have been overestimated in these two species. For *Avicennia marina*, the sap flow at the base of the live crown exceeded which at breast height (**Figures 4B, E**), indicating that part of the stem storage water was used for *E*_n_, while there was no *R*_n_ in the terminal branches. Thus, we can reliably estimate the contribution of *E*_n_ to *Q*_n_ in *Avicennia marina.*

In the water stress environment, mangrove species developed water-saving mechanisms to reduce water loss *via* transportation during the day (Ball, 1988). Our results indicated that a significant water compensation mechanism used by *Kandelia obovata* and *Aegiceras corniculatum* operated at night. Thus the more water was lost owing to diurnal transpiration, the higher the *Q*_n_ was at night to make up for the lost water. This newly found stratagem in mangrove species provides evidence of a nocturnal water recharging mechanism in addition to their diurnal water-saving strategies. We suggest that mangrove species with nocturnal water recharging mechanisms and tight control of stomata will be better adapted to future environmental conditions of enhanced atmospheric evaporation demand than species without these capabilities. As for the *Avicennia marina*, its unique daytime hydration and timely restriction of water loss under high salt conditions provides its ability to survive under conditions of limited water.

### Driving force of nocturnal sap flow in mangroves

*Q*_n_ is driven by environmental factors and biotic factors, separately or synergistically. Previous studies suggested that VPD was the main environmental factor driving *E*_n_ because VPD between the leaf surface and the atmosphere provided conditions for stomata to open or partially open at night (Caird et al., 2007; Dawson et al., 2007; Zeppel et al., 2010). Wind speed affected the moisture movement around plant canopy (Fricke, 2019) and thus influenced on nocturnal water loss (Chen et al., 2020), which also could not be ignored. In this study, we found that wind speed was not a driving force in sap flow, whether during the daytime or nighttime, which is consistent with the result that wind speed had little effects on the nocturnal water loss by Wang et al., 2018 and Wu et al., 2020. Rosado et al. (2012) found strong positive relationships between VPD and *Q*_n_ in woody species in rain forest and indicated that the *E*_n_ was driven by atmospheric demand for water. Wang et al. (2018) concluded that VPD had little effect on *Q*_n_ because the correlation coefficient was low (R^2 = ^0.100, *P*< 0.0001). Similarly, our findings also showed that VPD had only a slight effect on the *Q*_n_ of *Kandelia obovata* and *Aegiceras corniculatum*, indicating that VPD was not the main driver of *Q*_n_. In addition, there was a significant positive correlation between *Q* and *Q*_n_ in *Kandelia obovata* and *Aegiceras corniculatum*. Based on the principle of water balance, *Q*_n_ of these two species was thus mainly driven by the demands of stem water refilling after diurnal water depletion. In addition, high salinity conditions were also a driver of *Q*_n_ in *Kandelia obovata* and *Aegiceras corniculatum*, and the high salinity environment prompts both of the species to refilling more water at night. However, the *Q*_n_ of *Avicennia marina* occurred mainly in the early night and was driven by VPD (R^2 = ^50.3%) (**Figure S2**, **Table 2**). Our results suggest that *Q*_n_ is driven by VPD, high salinity conditions or the demand for stem rehydration, separately or synergistically. The main driving force differs among species.

## Conclusions

The *Q*_n_ of three co-occurring intertidal mangrove species (*Kandelia obovata*, *Avicennia marina*, and *Aegiceras corniculatum*) contributed markedly to daily total sap flow (5.5%–24.0%) across species during the study period. The diversity of stem recharge patterns and response to sap flow to high salinity conditions are the main reasons for the differences in *Q*_n_/*Q* among species. Different degrees of control of leaf stoma at night were found. *Kandelia obovata* and *Aegiceras corniculatum* exhibited strict control over their stomata to reduce water loss. In contrast, a large part of the *Q*_n_ of *Avicennia marina* was dissipated into the atmosphere through the stomata of the leaves. *Q*_n_ of three co-occurring mangrove species consisted of *E*_n_ and *R*_n_. *R*_n_ was the main contributor to the *Q*_n_ in *Kandelia obovata* and *Aegiceras corniculatum* (56.3% and 67.3%, respectively), which was an effective water-use strategy for avoiding physiological dehydration and xylem embolism. However, the *Q*_n_ of *Avicennia marina* was mainly used for *E*_n_ (*E*_n_ accounted for 103% of *Q*_n_), and the stem water storage was partially depleted at night during the nocturnal transpiration process. The unique daytime hydration and timely restriction of water loss under high salt conditions allow the *Avicennia marina* to adapt to harsh habitats. The major driver of *Q*_n_ in *Kandelia obovata* and *Aegiceras corniculatum* was the demand for stem water refilling after diurnal water depletion and high salinity, while that of *Avicennia marina* was VPD. Our findings highlight the diversity of nocturnal water use strategies in co-occurring mangrove species and the conservative nocturnal water use in mangrove species. The diverse ways *Q*_n_ properties act as water-compensating strategies among the co-occurring mangrove species will help mangrove species to adapt to future climate conditions.

## Data availability statement

The raw data supporting the conclusions of this article will be made available by the authors, without undue reservation.

## Author contributions

SW, XG, YZ, and LC planned and designed the research. SW, XG, and YZ performed experiments and conducted fieldwork. SW analyzed data. SW, XG, and LC contributed to writing the manuscript. All authors contributed to the article and approved the submitted version.
