# Supplementary material for: Nocturnal sap flow as compensation for water deficits: an implicit water-saving strategy used by mangroves in stressful environments
Source: Front Plant Sci. 2023 May 8;14:1118970. doi: 10.3389/fpls.2023.1118970 (PMC10200988; doi:10.3389/fpls.2023.1118970)
Supplement: Supplementary file 1 [file DataSheet_1.docx]

**Figure S1. The relationship between wind speed and sap flow in per sapwood area for each species from August to December.** *y*_1_ and *y*_2_ represented mean daily sap flow and mean nocturnal sap flow respectively.

**Figure S2.** **Variation of nocturnal sap flow density (SFD_n_) and vapor pressure deficit (VPD) at clear night.** The data were averaged from May 11-13, June 5-7, August 24-26 and September 7-10 in 2019. The means and standard errors of four replicates are shown.

**Fig. S1**


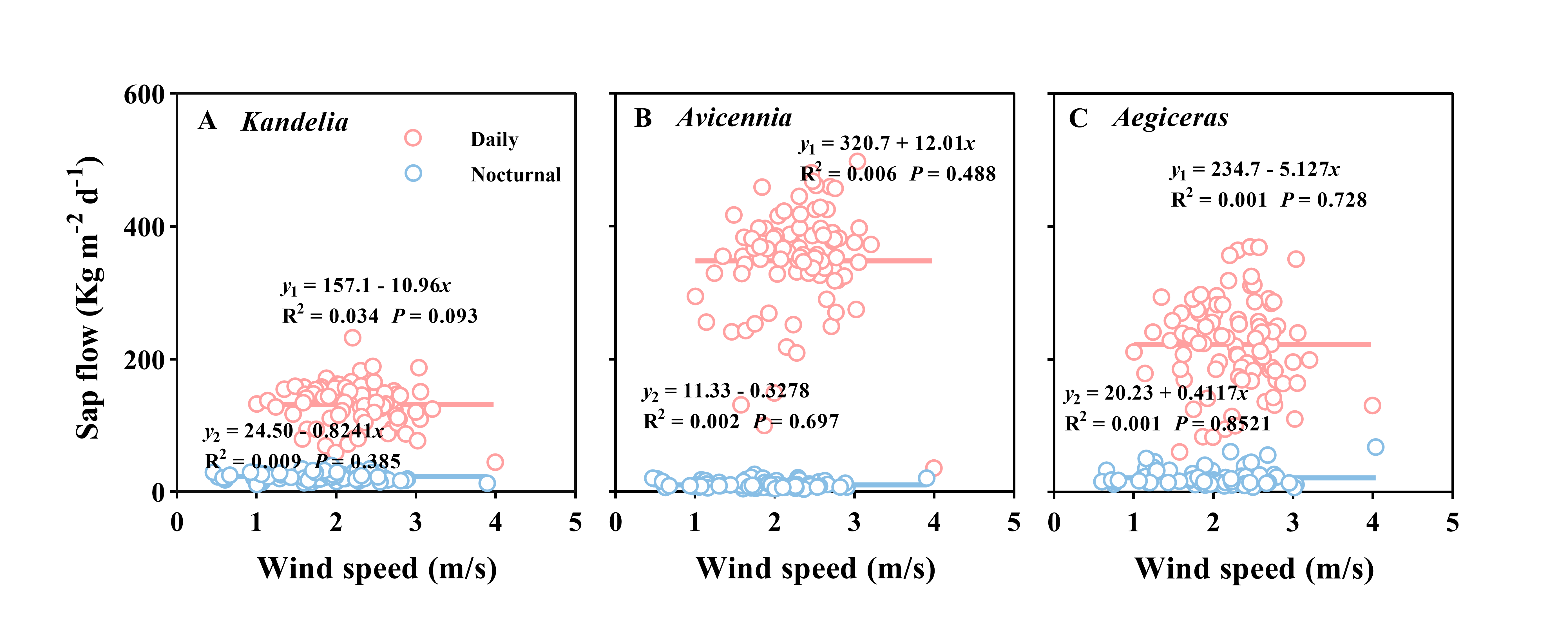


**Fig. S1 The relationship between wind speed and sap flow in per sapwood area for each species from August to December.** *y*_1_ and *y*_2_ represented mean daily sap flow and mean nocturnal sap flow respectively.

**Fig. S2**


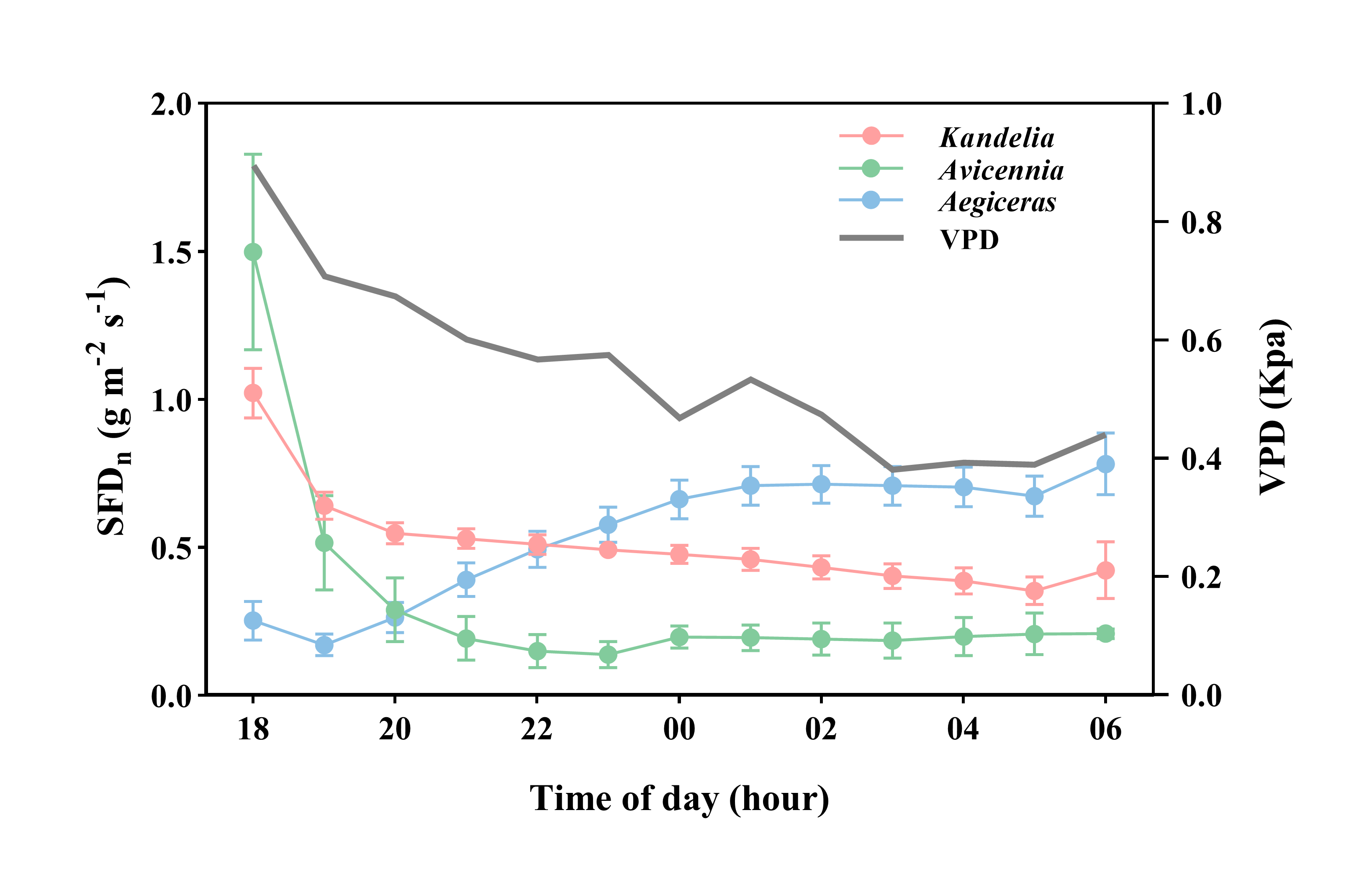


**Fig. S2** **Variation of nocturnal sap flow density (SFD_n_) and vapor pressure deficit (VPD) at clear night.** The data were averaged from May 11-13, June 5-7, August 24-26 and September 7-10 in 2019. The means and standard errors of four replicates are shown.
